# Body Composition, Lipid Profile, Adipokine Concentration, and Antioxidant Capacity Changes during Interventions to Treat Overweight with Exercise Programme and Whole-Body Cryostimulation

**DOI:** 10.1155/2015/803197

**Published:** 2015-06-15

**Authors:** Anna Lubkowska, Wioleta Dudzińska, Iwona Bryczkowska, Barbara Dołęgowska

**Affiliations:** ^1^Department of Functional Diagnostics and Physical Medicine, Faculty of Health Sciences, Pomeranian Medical University in Szczecin, 54 Żołnierska Street, 71-210 Szczecin, Poland; ^2^Department of Physiology, Faculty of Natural Sciences, Szczecin University, 3c Felczaka Street, 71-412 Szczecin, Poland; ^3^Department of Laboratory Diagnostics and Molecular Medicine, Pomeranian Medical University in Szczecin, 72 Powstańców Wielkopolski Avenue, 70-111 Szczecin, Poland

## Abstract

The aim of this study was to determine the effect of six-month-long physical exercise programme with a two-time exposure to whole-body cryostimulation (WBC) in 20 sessions on antioxidant enzyme activities, lipid profile, and body composition changes in obese people (30 adult subjects; BMI = 30.39 ± 4.31 kg/m^2^). Blood samples were taken before the programme, one month following the exercise programme, before and after the first WBC treatment, six months following the exercise programme, after the second WBC treatment, and finally one month after the intervention. Six months of moderate aerobic activity combined with WBC did not change body mass or fat and lean body mass percentages, or circulating adiponectin, leptin, and resistin concentrations. In response to intervention a significant decrease in the level of low-density lipoprotein and triglycerides was observed, with a slight increase in high-density lipoprotein concentration. The nature of changes in the activity of respective antioxidant enzymes was not identical. After one month of increased physical activity, a significant decrease in superoxide dismutase, catalase, and glutathione reductase activities was observed (13%, 8%, and 70%, resp.). The SOD activity increased significantly after successive whole-body cryostimulation sessions. As regards catalase, a significant progressive decrease in its activity was observed.

## 1. Introduction

Obesity results from excessive energy intake compared with the energy expenditure. The World Health Organisation (WHO) defines the weight status according to body mass index (BMI), that is, the ratio of weight in kilograms divided by height in meters squared. A BMI of 20 to 25.9 is considered a normal weight, 25 to 29.9 is overweight, and that equal to or greater than 30 is obesity [1].

The percentage of people living a sedentary lifestyle, which is accompanied by increased visceral fatty tissue deposit, being a source of hormones and adipokines and other biologically active factors, is still growing.

The clinical significance of abdominal obesity is well known and is associated with adverse changes in lipid indicators and its relationship with increased risk of coronary heart disease and hypertension [2]. Obesity in humans is considered a state of chronic inflammation, low-grade proinflammatory state, and adipocyte hypertrophy and hyperplasia leading to imbalanced adipokine secretion.

The increasing volume of adipocytes associated with development of adipose tissue inflammation and changes in leptin, resistin, tumour necrosis factor (TNF *α*), adiponectin, interleukin-6 (IL-6), plasminogen activator inhibitor-1 (PAI-1) secretion leads, consequently, to obesity-mediated adverse effects on glucose and lipid metabolism, development of insulin resistance, type 2 diabetes mellitus, and thrombosis or metabolic syndrome [3]. Numerous clinical studies show strong relationships between adipokines and body composition indices [4–7]. It has been reported that obesity may induce systemic oxidative stress (OS) and, in turn, OS is associated with irregular production of adipokines. Oxidative stress can result from either excess reactive oxygen species (ROS) production or deficient antioxidant capacity. On the other hand, low concentrations of free radicals, ROS, and other nitrogen species are necessary for normal cellular redox status, cell function, and intracellular signalling. The rates of free radical formation are increased in obesity and, moreover, oxidative stress is presently accepted as a likely causative factor in the development of insulin resistance [8]; in contrast, it is postulated that antioxidant defence markers are lower according to the amount of body fat and central obesity [9, 10]. BMI is significantly related to the concentration of F-2 isoprostanes (F2-IsoPs) and plasma malondialdehyde (MDA), being a product of polyunsaturated fatty acid peroxidation, while the activity of superoxide dismutase (SOD) and glutathione peroxidase (GPx) in people with obesity is significantly lower compared with that in slim persons [11, 12]; moreover, 8-isoprostanes have been identified as markers of oxidative stress in obese patients with heart disease and are strongly associated with visceral than with subcutaneous adipose tissue. Abdominal or visceral adiposity is linked to excessive intracellular triglycerides and dyslipidaemia as well as elevated plasma free fatty acids. Hypercholesteraemia is associated with enhanced oxidisability of low density lipoprotein (LDL-C) molecules. It is well established that exercise training lowers the risk of developing cardiovascular disease and has a positive effect on body weight and cardiovascular disease risk factors in people with overweight or obesity. Physical activity, dietary restriction, and surgical interventions reduce oxidative stress levels. Regular and properly dosed physical exercise leads to reduction of visceral fat deposit and improvement of adipokine secretion.

Even if physical exercise does not result in weight loss, it confers significant health benefits to people with overweight and obesity, reduces the risk of developing hypertension increasing high-density lipoproteins, improves myocardial antioxidant capacity, and may also stimulate the immune function of organism [13]. Whole-body cryostimulation is a brief exposure, well tolerated by the human body, to very cold air (−130°C) in a cryochamber for 2-3 minutes causing active peripheral hyperaemia, which results in improved metabolism. Anti-inflammatory, pain relieving, and positive effects of cryogenic treatments on the immune and endocrine systems are also often indicated [14]. It is postulated that cryostimulation stimulates antioxidant potential and enhances favourable adaptation mechanisms. Our previous study has shown that even a single cryostimulation modifies the level of plasma 8-isoprostanes and both enzymatic and nonenzymatic antioxidants in the body [15, 16]. Moreover, after 20 daily WBC treatments, an increase in SOD and SOD : CAT ratio has been observed in normal-weight people, with a decrease in reduced and oxidised glutathione concentration and GPx activity [16]. A positive effect of low temperatures on the mental state has also been observed [17]. In the light of the above findings, the objective of this study was to assess the effect of six-month-long physical exercise programme with whole-body cryostimulation as a means to change body composition, lipid profile, adipokine concentration (adiponectin, leptin, visfatin, and resistin), and antioxidant enzyme activity in people with overweight and obesity. As regards prooxidant-antioxidant equilibrium, our analysis focused on the changes in plasma 8-isoprostane concentration (marker of oxidative stress) and superoxide dismutase 1 (Cu/Zn-SOD, EC 1.15.1.1), catalase (CAT, EC 1.11.1.6), glutathione peroxidase (GPx, EC 1.11.1.9), glutathione reductase (GSSG-R, EC 1.8.1.7), and glutathione S-transferase (GST, EC 2.5.1.18) activities.

## 2. Material and Methods

### 2.1. Subjects and Lifestyle Interventions

The study was approved by the Local Ethics Committee of the Pomeranian Medical University and the Bioethics Committee of the Regional Chamber of Physicians and Dentists in Kraków.

This study was performed in compliance with the Declaration of Helsinki as well. Examinations were limited to adult subjects. Forty-five overweight and obese men participated in the experiment (40 ± 4.0 years of age, height of 178 ± 3.0 cm, BMI = 30.39 ± 4.31 kg/m^2^). Prior to the experiment, each participant was examined by a physician to test for any contraindications towards cryostimulation and maximal physical effort. Exclusion criteria included a history of cardiovascular disease, stroke or transient ischaemic attacks, diabetes mellitus, blood pressure > 160/100 mmHg, total cholesterol > 300 mg/dL, smoking, active participation in any exercise activity ≥ 30 min more than once a week, participation in organised diet programmes, and heart, renal, or liver disease.

For each subject, we performed basic anthropometric measurements (body weight, body height, waist and hip circumference, and waist to hip ratio (WHR)). Resting blood pressure (BP) was measured 3 times in a seated position using a mercury sphygmomanometer. The average of these 3 readings was used as the representative examination value. The measurement was performed under controlled conditions in a quiet room. An electrical bioimpedance method (using a Jawon Medical X-Scan Plus II body composition analyser) was used to estimate body composition parameters: body fat mass (BFM), subcutaneous fat mass (SFM), visceral fat mass (VFM), lean body mass (LBM), skeletal muscle mass (SMM), and the percentage of these components during the study. One week before the treatment, the participants were subjected to a progressive ergocycle test with incremental intensity until exhaustion, establishing their maximal oxygen uptake (VO_2max_) to assess aerobic capacities.

The exercise was preceded by a 5 min warm-up (25 W). The test was performed on a bicycle ergometer (Monark 839E), beginning with a workload of 1 W per kg of fat-free mass (1 W kg_FFM_^−1^) that was increased by half of this value (0.5 W kg_FFM_^−1^) every 3 min until volitional exhaustion. The VO_2max_ criterion represented the achievement of expected maximal heart rate (HRmax) and a lack of increase in oxygen uptake (VO_2_) despite the increasing workload. During the exercise, oxygen uptake (VO_2_) was measured continuously using an Oxycon gas analyser (Jaeger, Germany). The same procedure of VO_2max_ assessment was repeated after 6 months of intervention.

Intensive lifestyle modification programme consisted of a structured 6-month-long exercise programme being developed by an exercise physiologist, but without diet modification. The exercise programme consisted of moderate-intensity progressive cardiovascular exercise involving both the upper and the lower limbs. The subjects exercised 3 times a week (45 minutes each), twice a week in the gym under close supervision of an exercise physiologist, and once a week a Nordic walking session in an open area. Participants progressed training from 50% to 75% of maximum heart rate. Heart rate monitors, Polar S610 (Polar, Finland), were used to adjust workload to achieve the target heart rate. Facility sessions consisted of stationary bicycling, aerobics, step-up exercise using a 20-cm high bench, strength training elements consisting of 2 sets of 10 repetitions of leg extension, leg curls, and leg press. All participants of the training group stretched before and after each training session. After a period of one month from the start of the fitness programme, whole-body cryostimulation procedure was implemented, with a scheme of 20 daily treatments (in the second month of the exercise programme). Application of these treatments was repeated, with the same scheme, in the last month of the study. The subjects were instructed to maintain their typical diet and activity pattern throughout the study. There was no change in calorie intake or diet plan.

**Figure 1:**
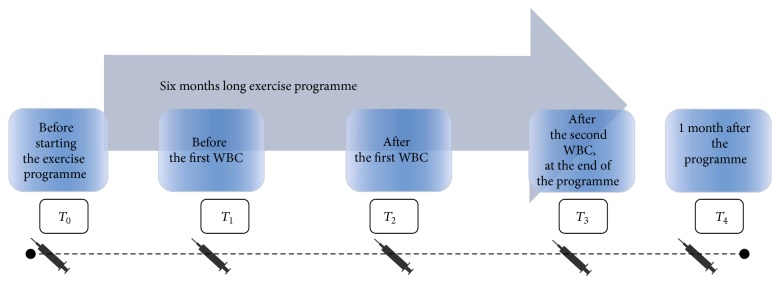
Schedule of the experiment.

It should be noted that 30 subjects, whose results were analysed, lasted out until the end of the exercise programme. Other participants dropped out of the study due to different reasons, not related however to health deterioration or injury occurrence. The course of the study, including WBC as well as blood sampling and anthropometric measurements, is presented in Figure 1.

### 2.2. Cryostimulation Procedure

Systemic cryotherapy procedure was conducted by means of 20 daily entries into a cryochamber. The treatments were performed every day, five days a week, Monday through Friday, at the same time in the morning. The subjects entered the chamber in groups of four. Each session lasted 2-3 minutes at −120°C. Each entry to the cryochamber was preceded by a 30-second adaptation period in the vestibule at −60°C. After adaptation, the subjects went further into the proper chamber, where they slowly moved in a circle, one after the other; no additional movement, mutual contact, or talking was permitted. The total time a subject stayed in the cryochamber was not longer than 3 minutes.

A change in the motion direction was recommended after 1 minute. Each subject was informed about shallow breathing (short inhalation and longer exhalation). Regular contact with the subjects was maintained via a camera and a voice system in the room. Before each treatment, systolic and diastolic blood pressures were measured to monitor one of the contraindications towards cryostimulation, that is, high blood pressure. Glasses, contact lenses, and all jewellery were removed before entering the chamber, and the body was thoroughly dried to eliminate the sensation of cold, and noses and mouths were secured with a surgical mask. During the cryostimulation procedure, the subjects wore only shorts, socks, wooden clogs, gloves, and a hat covering the auricles against frostbite.

### 2.3. Blood Sampling and Biochemical Analysis

During the experimental period, venous blood samples were collected five times to perform haematological and biochemical analyses: *T*_0_, before the exercise programme; *T*_1_, one month following the exercise programme, before the first WBC treatment; *T*_2_, after the first WBC treatment; *T*_3_, six months following the exercise programme, after the second WBC treatment; and *T*_4_, one month after the intervention. Each time, venous blood samples were obtained in the morning after an overnight fasting, between 8:00 and 9:30 a.m., from the antecubital forearm vein, after a 10-minute rest in the sitting position, using the Vacutainer system tubes with appropriate anticoagulant (Sarstedt, Germany), for biochemical analysis of erythrocytes and plasma (7 mL; EDTA) and determination of blood count (1.2 mL; anticoagulated with 1 g/L EDTA): erythrocyte number (RBC), haemoglobin concentration (Hb), haematocrit value (Ht), leukocyte number (WBC), and thrombocyte number (PLT). Haematological parameters were measured using an automated haematology analyser (HORIBA ABX Micros 60), while another one was used in the biochemical analysis of serum (5 mL). Erythrocytes were separated by centrifugation (300 rpm, 1500 g, 10 min, 4°C), washed three times with a buffered NaCl solution (PBS: 0.01 mol phosphate buffer, 0.14 mol NaCl, pH 7.4), chilled to 4°C, and finally frozen at −70°C. Plasma was divided into aliquots and immediately deep-frozen at −70°C until analysis, but not longer than one month. A spectrophotometric method was used to determine glucose, uric acid, total cholesterol, high-density lipoprotein (HDL-C), and triglycerides (TG). In addition, the concentration of LDL cholesterol fraction was determined using the Friedewald formula:

  LDL (mg/dL) = total cholesterol (TCH) − HDL-cholesterol − (TG/5) [18].

Commercial enzyme-linked immunosorbent assay (ELISA) kits were used to measure serum 8-isoprostane levels (Cayman, MI, USA), according to the manufacturer's protocol.

### 2.4. Analysis of Erythrocyte Antioxidative Enzyme

Before the analysis, erythrocytes were thawed and the resulting haemolysate of the washed red blood cells was diluted with distilled water and chilled to 4°C. The SOD, CAT, GPx, R-GSSG, and GST activities were measured in the haemolysate samples with a BIOXYTECH kit (Oxis Research, Portland, OR, USA) using a UV/VIS Lambda 40 spectrophotometer (Perkin-Elmer, Wellesley, MA, USA); SOD: sensitivity: 0.1 U/mL; specificity: 97%; coefficient of variation: lower than 4%; CAT: sensitivity: 1.71 U/mL; specificity: 89%; coefficient of variation: lower than 2%; GPx: sensitivity: 6 mU/mL; specificity: 94%; coefficient of variation: lower than 4%, GSSG-R: sensitivity: 0.14 mU/mL; specificity: 94%; coefficient of variation: lower than 4%; GST: sensitivity: 1.2 mU/mL; specificity: 94%; coefficient of variation: lower than 4%.

The enzyme activities were calculated per 1 g of erythrocyte haemoglobin. In all mentioned cases, haemoglobin levels were assayed using the Drabkin method [19]. The serum levels of adiponectin, leptin, visfatin, and resistin were measured by immune-enzymatic assays using commercially available ELISA kits (R&D Systems, Abingdon, UK), according to the manufacturer's instructions. The adiponectin assay sensitivity was 0.246 ng/mL, while intra- and interassay coefficients of variation (CV) were 2.5–4.7% and 5.8–6.9%, respectively. The leptin assay sensitivity was 7.8 pg/mL, and intra- and interassay CVs were 3.3–3.2% and 4.2–3.5%, respectively.

### 2.5. Statistical Analyses

The obtained results were statistically analysed. Distributions were examined using the Shapiro-Wilk test which indicated that some variables deviated from a normal distribution (they were lognormal). Each studied parameter was characterised by sample size, arithmetic mean/median, and standard deviation. The data were tested by one-way ANOVA. Since in some cases the distribution was not normal, a Friedman post hoc test and a nonparametric Wilcoxon post hoc test for a dependent variable were performed when a significant *F*-value was found. The accepted level of significance was defined as *p* < 0.05. Development of the statistical results was performed using STATISTICA PL v 7.1 software (Statsoft, Kraków, Poland). Statistical significance was assumed at *p* < 0.05.

## 3. Results

Table 1 presents the basic anthropometric and physiological parameters of the examined group at the start of the training period. Based on this assessment, we found that all subjects were overweight or obese. The average value of BMI was 30.39 ± 4.31 kg/m^2^ (between 26.3 and 41.1 kg/m^2^), while the waist/hip ratio ranged 0.84–1 (mean value 0.88 ± 0.05). According to the European Society of Hypertension (Guidelines Committee, 2003) [20], all of them, except two, were normotensive. The mean (mean ± SD) resting systolic pressure was 131 ± 10 mmHg, while diastolic pressure 78 ± 6 mmHg.

The subjects were characterised by a high percentage of adipose tissue, both subcutaneous (30.56 ± 4.03%) and visceral (4.96 ± 0.93%).  Table 2 shows the changes in body composition parameters during the exercise programme. It could be noticed that a 6-month-long exercise programme did not change body weight or BMI, or WHR, although a downward trend was observed. Similarly, no significant changes were observed in the content of respective body components during the exercise programme, both in relation to their percentage and absolute values. A downward trend, appearing after the first month of physical activity, can be seen in relation to some nonfat components (MBF, SFM, and VFM), while an upward trend in relation to other ones (LBM, SSM), but without statistically significant differences.

**Figure 2:**
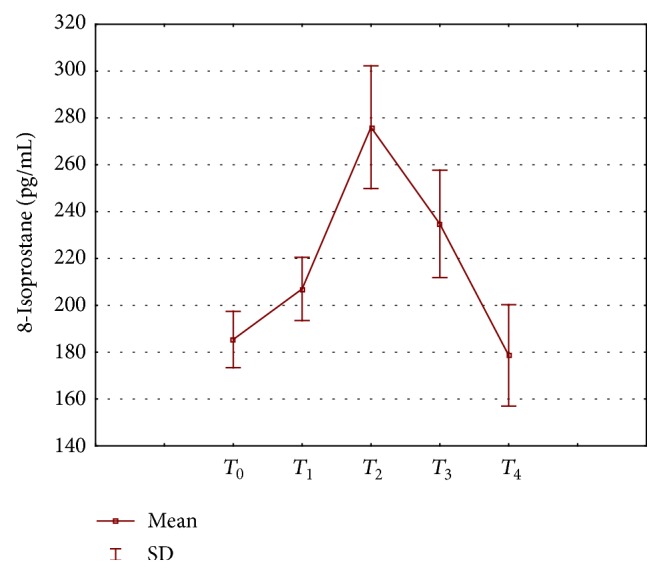
Changes in plasma concentration of the 8-isoprostane during the exercise programme.

**Figure 3:**
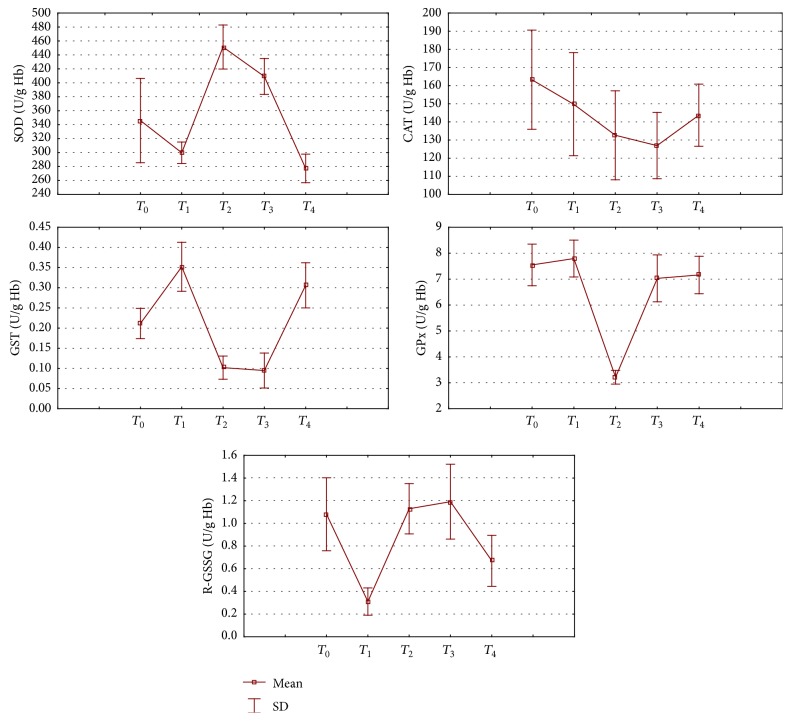
Changes in superoxide dismutase (SOD), catalase (CAT), glutathione S-transferase (GST), glutathione peroxidase (GPx), and glutathione reductase (GSSG-R) activity in erythrocytes of subjects following the exercise programme.

The exercise programme caused only a small, insignificant increase in aerobic capacity (maximal oxygen uptake; Table 1). For all subjects, the baseline values of all haematological indices were within clinical and laboratory reference values. In the first month of the exercise programme, no changes were observed in the values of red and white blood cell parameters, whereas in the next month, after application of the first series of whole-body cryostimulation treatment, a significant decrease was observed in red blood cell count (to 3.87 ± 1.04 × 10^12^/L), haemoglobin concentration (to 7.08 ± 1.11 mmol/L), and haematocrit value (to 0.33 ± 0.07 L/L). Significantly lower values of these parameters, below baseline ones, were maintained until the end of the exercise programme, although they were already showing upward trends, reaching a level similar to the baseline one month after the end of the study. The observed changes were accompanied by an increase in mean corpuscular haemoglobin (MCH), mean corpuscular haemoglobin concentration (MCHC), and red blood cell distribution width (RDW). As regards white blood cell parameters, only a temporary decrease in white blood cell count was observed in the last month of the study, to 6.49 ± 1.26 × 10^9^/L (Table 3). Studies on lipid changes in response to exercise are summarised in Table 4. The participants were characterised by unfavourable high baseline values for total cholesterol concentration (205.91 ± 37.64 mg/dL), LDL-C (146.31 ± 32.76 mg/dL), and TG (125.91 ± 56.71 mg/dL), with relatively low concentration of high-density lipoprotein (HDL-C = 29.88 ± 5.27 mg/dL) at the same time. During the exercise programme, a significant decrease was observed in the level of low-density lipoprotein (LDL-C) and triglycerides (TG) after the first month of physical activity, being maintained throughout the course of the exercise programme but returning to high values, slightly exceeding the baseline, one month after stopping the physical activity. After six months of the exercise programme, total cholesterol concentration also was temporarily reduced (below 200 mg/dL) but this decrease was not maintained in the month following the study. The changes observed in the lipid profile were accompanied only by slight alterations in the concentration of HDL-C, with an upward trend being observed after the first and the second series of whole-body cryostimulation. When considering the serum level of adipokines, only a significant increase in serum visfatin concentration was observed, from the baseline value of 0.77 ± 0.5 ng/mL to 1.47 ± 0.6 ng/mL after the end of physical activity, being still maintained at an elevated level (1.37 ± 0.55 ng/mL) one month after the end of the exercise programme. Adiponectin, leptin, and resistin levels did not change during the study.  Figure 2 shows a change in the level of isoprostanes during the exercise programme. A significant increase in their concentration was observed after the first month of physical activity, from 185.05 ± 23.43 to 207.02 ± 34.83 pg/mL (*p* = 0.0318); moreover, we recorded a significant increase, up to 50%, in their concentration in response to incorporated series of cryostimulation treatment, to 276.02 ± 47.31 pg/mL. It is noteworthy that, one month after the exercise programme (*T*_4_), the lIso-8 level was reduced to a value slightly lower than the baseline (178.6 ± 43.57 pg/mL). When considering the whole-body antioxidant potential, changes were observed in the activity of respective enzymes. After one month of increased physical activity, a significant decrease was observed in the activity of superoxide dismutase (13%), catalase (8%), and glutathione reductase (70%). It should be noted that the nature of changes in the activity of respective antioxidant enzymes during successive months of the exercise programme was not identical. After an initial reduction, the SOD activity significantly increased during *T*_2_ and *T*_3_, that is, after successive cryostimulation series, but then decreased below the baseline values one month after the exercise programme. As regards catalase, a progressive significant decrease was observed in its activity, reaching 22% after the second WBC series, but then it started to grow after the end of the intervention, not reaching however the baseline values. Changes of the opposing nature were observed for GST and R-GSSG; the GST activity decreased and that of R-GSSG returned to the baseline values after an initial decrease following the application of whole-body cryostimulation. The changes observed in the activity of analysed antioxidant enzymes are presented on graphs in Figure 3.

## 4. Discussion

The focus of the study was to determine the changes in body composition, lipid profile, adipokine serum level, and antioxidant capacity in obese people following a six-month-long exercise programme, including two cryostimulation treatments as 20 daily sessions in the second and the last month of intervention, without diet modification. Recommendations for the treatment of adults who are overweight or obese focus on energy balance with lifestyle modifications designed to reduce daily energy intake and increase physical activity [21]. Whole-body cryostimulation, by using very low temperatures (ranging from −100°C to −160°C) over a short time period (1–3 minutes), induces systemic physiological responses and can be a supplementary method for obese persons in reducing body mass and systemic inflammation [22]. The adipose tissue serves as a store of readily mobilised substrate, free fatty acids (FFA), for calorigenesis in other tissues during cold exposure, principally for muscular shivering thermogenesis. Lipid metabolism, including lipogenesis, lipolysis, and fatty acid oxidation, is under tight control of the sympathetic nervous system (SNS) and adipose-derived hormones [23]. Cooling of the skin during cryostimulation treatment activates cold-sensitive low- and high-threshold receptors subserved by A*δ* and C fibres with selective autonomic responses [24]. It seems that changes in lipid metabolism, taking place during repeated exposure to cryogenic temperatures applied on the whole body, may affect the concentration of respective lipid fractions in the blood serum of the subjects undergoing treatments and induce changes of the adaptive nature, the consequences of which could be of importance in the prevention of overweight and obesity and such diseases as metabolic syndrome or diabetes mellitus. Potential benefits and the changes taking place in the body under the influence of physical training depend on exercise intensity, activity duration, and fitness level [25]. In our study, exercises were moderate and conducted 3 times/week for six successive months, taking into account the potentially low level of physical capacity of the subjects. It has been observed in a longitudinal intervention study performed on 30 obese female and male subjects (BMI > 25 kg/m^2^, over 30% body fat) that exercise training programme did not change body mass significantly, neither the content of any of the body components, both fat and nonfat ones. It should be noted that no dietary restriction was applied to the subjects; therefore, it is very likely that they could increase the caloric content of their diet due to increased energy expenditure associated with the inclusion of physical activity into their lifestyle, which would give no effects related to the reduction of body mass. Despite the absence of weight loss, it is worth noting at the same time that a six-month intervention has been found to produce favourable changes in the lipid profile, as well as a decrease in triglycerides and LDL cholesterol, with a slight increase in HDL cholesterol, after WBC treatment. Plasma lipid levels are associated with the risk of coronary heart disease and it is documented that highly trained athletes have lower levels of total cholesterol and LDL cholesterol and higher levels of HDL cholesterol than sedentary persons of the same age and sex; however, prospective studies on exercise and lipid changes have yielded conflicting results. Some studies have reported that blood lipids are influenced more by diet treatments than exercise [26]. Obesity is accompanied by chronic oxidative stress, although there are no data in the available literature which would clearly explain whether obesity leads to chronic oxidative stress or whether chronic oxidative stress is one of the etiological factors of obesity. At the same time, it has been demonstrated that oxidative stress may be intensified by strenuous physical exercise, advanced age, and concomitant diseases. Numerous studies have shown an augmented lipid peroxidation during physical exercise because of increased oxygen consumption by the working muscles, which increases the production of superoxide anion radical initiating the oxidative stress and impairing the efficiency of antioxidant system. However, variation in the prooxidant-antioxidant equilibrium (PAE) during the physical activity depends on the nature and the intensity of exercise. Endurance and moderately intense exercises, being in addition gradually intensified, activate the antioxidant defence [27–29]. An additional factor being applied in our exercise programme was the whole-body cryostimulation. After exposure to extremely low temperature, the body experiences significant haemangiectasis (angio-osteodystrophy) and increased blood supply to internal organs, which leads to an increase in muscle oxygen concentration [30–32]. While only few studies on the effect of cryostimulation on oxidative and antioxidative processes in the cells of ill and healthy individuals have been published, it is suggested that repeated cryostimulation sessions may cause adaptive changes, for example, an increase in the antioxidant capacity [16, 33, 34]. Alterations in the metabolic activity and oxygen consumption during cold exposure may induce changes in the production of reactive oxygen species. It has been documented that even one WBC session resulted in a significant increase in plasma Iso-P level and further daily sessions sustained the elevated isoprostane, while WBC continuation for 20 sessions elevated the SOD activity up to 43%, which entailed a significant increase in the SOD : CAT ratio [16]. Importantly, the beneficial nature of biochemical changes (e.g., in lipid levels, anti-inflammatory cytokine level) was visible only after a longer series of exposures (20 sessions) [32, 35]. The present results are consistent with our earlier reports on WBC as a stress-inducing factor that activates favourable adaptation mechanisms and increases the resistance of the human body. The changes in plasma 8-isoprostane concentration (a metabolite of lipid peroxidation that serves as a time-integrated marker of oxidative stress) were observed during the exercise programme with cryostimulation. The first significant increase was provoked by repeated exercises; more importantly, cryostimulation sessions intensified the oxidative stress and it seems that free radical production exceeded antioxidant protection. To protect against oxidative stress, the changes occurred in antioxidant defence mechanisms to reduce the risk of oxidative damage during the periods of treatment. When comparing the activity of superoxide dismutase and R-GSSG during the training accompanied by cryostimulation, a similar nature of changes has been observed after one month of moderate-intensity training, with the activity of these enzymes being significantly decreased; then it significantly increased for SOD in the period following the application of whole-body cryostimulation, while R-GSSG returned to the baseline values. An opposite relationship has been observed for GST; after an increase in its activity in the first month of training, a significant, 52%, decrease below the baseline value was observed after inclusion of cryostimulation series, being also maintained after the second treatment series. There are not many reports on the impact of WBC on the peroxidant and antioxidant status. Miller et al. [36] have shown an increase in total antioxidant status, SOD activity, and uric acid level in plasma in multiple sclerosis patients submitted to 10 sessions of cryostimulation. In the presented study, a successive decrease in the CAT activity was observed during the whole exercise programme, reaching the maximum value after the second cryostimulation series. The first line of defence, specifically against the formation of hydroxyl radicals, involves the activity of superoxide dismutase, catalase, and glutathione peroxidase. As the result of the two-step dismutation of superoxide anion (^•^O_2_^−^) with the participation of SOD, hydrogen peroxide is produced (H_2_O_2_), which may be neutralised in the reaction of disproportionation with CAT and reduction with GPx [37, 38].

It is noteworthy that, after the first cryostimulation treatment, a consequent deep decrease in glutathione peroxidase (GPx) occurred, associated with a crucial increase in the SOD activity. GPx catalyses the reduction of H_2_O_2_ or organic hydroperoxide (ROOH) to water (H_2_O) and alcohol (ROH), respectively, using reduced glutathione (GSH) or, in some cases, thioredoxin or glutaredoxin as the electron donor. Although CAT and GPx share common substrates, CAT has a much lower affinity for H_2_O_2_ at low concentrations compared with GPx. GPX isoenzymes reduce a wide range of hydroperoxides ranging from H_2_O_2_ to complex organic hydroperoxides, being an important antioxidant to protect against ROS-mediated damage to membrane lipids and proteins [39].

Some of the data suggest that, in the absence of weight loss, exercise training alone does not improve the adipokine profile or the levels of oxidative stress in overweight children [40]. According to the adipokine profile, only visfatin serum level was significantly elevated in our study and remained at a high level throughout the whole intervention.

It seems that the reason for no significant alteration in the adipokine levels in this study was indeed the absence of changes in visceral as well as subcutaneous adipose tissue which could be the result of low-intensity type of exercise protocol. Visfatin is an adipokine being mainly produced in visceral adipose tissue, also by cells, neutrophils and macrophages, and, furthermore, its plasma level correlates with the quantity of visceral fat in humans [41, 42]; however, the studies examining the effect of exercise on circulating visfatin levels are limited and often ambiguous. Choi et al. have noticed that exercise training resulted in weight loss and induced a significant reduction in plasma visfatin levels in young overweight Korean women [43], whereas Haider et al. have demonstrated that exercise training could even lower plasma visfatin concentration in patients with type 1 diabetes mellitus [44]. On the other hand, Büyükyazı et al. have found no significant change in the visfatin concentration in obese women (30–49 years) in moderate-intensity walking programme [45]. Pagano et al. have reported that visfatin levels were lower in subcutaneous fat locations and higher in visceral adipose tissue of the obese subjects compared to lean individuals [46]; on the contrary, Berndt et al. have not reported any significant relationships between visfatin levels and the amounts of visceral adipose tissue as determined by CT measurements [47]. A positive correlation between visceral adipose tissue visfatin gene expression and body mass index (BMI) has been found, but the relationship between subcutaneous fat visfatin and BMI was negative suggesting that visfatin regulation may differ depending on different fat patterns [48]. It could be concluded that changes in circulating visfatin levels are involved in the improvement of metabolic status by exercise and may be useful markers for exercise evaluation and prescription.

In the case of obese people, low- or moderate-intensity exercise training is recommended, associated with a loss of 150–200 kcal in 30 minutes, when the percentage of free fatty acids covering the energy requirements is the highest [49]. The efficiency of physical exercise in reducing the body mass depends on both its intensity and duration of exercise loads. The current guidelines of the American College of Sports Medicine (ACSM) and the American Heart Association (AHA) on preventive treatment of civilisation-related diseases for healthy people aged 18–65 years include a minimum 30-minute moderate endurance exercise at least 5 times a week [50], which often is difficult to be achieved and maintained in a long time due to, among other things, significant time-related and organisational interference as well as lack of interest in physically active lifestyle being frequently observed in overweight and obese people. Based on the performed study, it is possible to conclude that the efficiency of exercise programmes related to body mass reduction in people with overweight and obesity should be based on the modification of lifestyle being associated with an increased level of physical activity, while taking into account the dietary modification. Additional benefits and diversification of lifestyle modification result also from the inclusion of physical impact with confirmed whole-body effect into the exercise programmes, the example of which can be whole-body cryostimulation, but in compliance with all the rules and knowledge of its application.

Perhaps, it should be considered that combined resistance and aerobic exercise training or high-intensity interval training could be better than aerobic exercise alone to improve metabolic indicators. Several factors other than exercise may have influenced the results of this study. Low motivation and perseverance might be an intrinsic problem in influencing the lifestyle of obese people. This fact should be taken into account in future studies.

## Figures and Tables

**Table 1:** Anthropomorphological parameters in the study group at the start of the training period.

Parameters	*n* = 30		
	Mean value	Min	Max
Age [years]	39.06 ± 9.36	23	54
Body weight [kg]	87.3 ± 18.46	61	135
Body height [cm]	168.76 ± 8.24	153	192
BMI [kg/m^2^]	30.39 ± 4.31	26.3	41.1
WHR	0.88 ± 0.05	0.84	1
SBP [mmHg]	131 ± 10	110	151
DBP [mmHg]	78 ± 6	70	95
LBM			
[kg]	56.18 ± 12.32	40.6	96.5
[%]	64.48 ± 4.76	55.99	73.8
MBF			
[kg]	31.13 ± 8.56	20.4	55.4
[%]	35.43 ± 4.62	26.2	43.2
SFM			
[kg]	26.71 ± 6.99	18.00	46.10
[%]	30.56 ± 4.03	22.13	36.62
VFM			
[kg]	4.42 ± 1.6	2.40	9.30
[%]	4.96 ± 0.93	3.52	7.39
SMM			
[kg]	25.1 ± 7.66	13.3	50
[%]	28.72 ± 6.19	19.11	41.51
VO_2max_ *T*_0_ [mLkg^−1^ min^−1^]	42.76 ± 4.2	39.41	49.31
VO_2max_ *T*_3_ [mLkg^−1^ min^−1^]	44.21 ± 7.1	40.3	51.1

BMI: body mass index; WHR: waist : hip ratio; SBP: systolic blood pressure; DBP: diastolic blood pressure; LBM: lean body mass; MBF: body fat mass; SFM: subcutaneous fat mass; VFM: visceral fat mass; SMM: skeletal muscle mass.

**Table 2:** Body composition changes following the exercise programme.

Parameters	*T*_0_		*T*_1_		*T*_3_		*T*_4_	
	$\overset{-}{x}$	Min–max	$\overset{-}{x}$	Min–max	$\overset{-}{x}$	Min–max	$\overset{-}{x}$	Min–max
Body weight [kg]	87.3 ± 18.46	61–135	83.5 ± 15.93	56.5–131.1	84.63 ± 18.81	54.3–128.1	85.66 ± 19.68	53.6–128.2
WHR	0.88 ± 0.05	0.84–1	0.87 ± 0.05	0.79–1.02	0.87 ± 0.05	0.78–0.99	0.87 ± 0.05	0.79–0.97
BMI [kg/m^2^]	30.39 ± 4.31	23.3–41.1	29.14 ± 3.73	22.9–36.7	29.43 ± 4.47	22.3–40.2	29.39 ± 4.94	21.7–38.3
MBF [kg]	31.13 ± 8.56	20.4–55.4	28.93 ± 7.47	15.2–49.8	29.17 ± 8.44	14.3–51.4	29.4 ± 9.18	14.6–51.1
MBF [%]	35.43 ± 4.62	26.2–43.2	34.65 ± 4.69	25.9–42.8	34.34 ± 5.17	22.6–42	34.15 ± 5.73	22.1–43
SFM [kg]	26.71 ± 6.99	18–46.1	25.03 ± 6.1	13.7–42.4	25.16 ± 6.88	13–43.3	25.35 ± 7.51	13.2–42.9
SFM [%]	30.56 ± 4.03	22.13–36.62	29.95 ± 3.96	22.26–36.46	29.72 ± 4.3	19.47–35.63	29.55 ± 4.75	19.19–36.14
VFM [kg]	4.42 ± 1.6	2.4–9.3	4.01 ± 1.37	1.5–7.4	4.01 ± 1.60	1.3–8.1	4.06 ± 1.69	1.4–8.2
VFM [%]	4.96 ± 0.93	3.52–7.39	4.71 ± 0.92	2.66–6.36	4.62 ± 1.06	2.39–6.58	4.61 ± 1.14	2.61–6.91
LBM [kg]	56.18 ± 12.32	40.6–96.5	54.46 ± 10.83	41.3–83.7	55.46 ± 12.96	40–93.3	56.25 ± 13.94	39–99.9
LBM [%]	64.48 ± 4.76	55.99–73.8	65.34 ± 4.69	57.18–74.05	65.67 ± 5.18	57.96–77.43	65.84 ± 5.75	56.95–77.93
SMM [kg]	25.10 ± 7.66	13.3–50	23.84 ± 6.98	13.4–38.6	26.21 ± 7.99	16–48.5	26.33 ± 9.72	14.1–61.2
SMM [%]	28.72 ± 6.19	19.11–41.51	28.57 ± 6.49	19.03–37.78	30.99 ± 6.14	21.88–45.03	30.53 ± 6.66	18.77–47.74

**Table 3:** Hematological indices changes following the exercise programme.

Parameters	*T*_0_		*T*_1_		*T*_2_		*T*_3_		*T*_4_	
	$\overset{-}{x}$	Min–max	$\overset{-}{x}$	Min–max	$\overset{-}{x}$	Min–max	$\overset{-}{x}$	Min–max	$\overset{-}{x}$	Min–max
RBC [10^12^/L]	4.84 ± 0.44	3.85–5.89	4.88 ± 0.40	4.19–5.65	3.87 ± 1.04 ^*∗∗∗*^ *T*_0_, *T*_1_	2.83–8.69	4.41 ± 0.34 ^*∗*^ *T*_0_, *T*_1_, ^*∗∗*^ *T*_2_	3.83–4.96	4.76 ± 0.34 ^*∗∗∗*^ *T*_2_	4.23–5.28

HGB [mmol/L]	7.60 ± 0.73	6.5–8.8	7.67 ± 0.67	6.60–9.4	7.08 ± 1.11 ^*∗*^ *T*_0_	5.10–11.4	7.02 ± 0.75 ^*∗*^ *T*_0_	4.70–8.5	7.13 ± 0.75 ^*∗*^ *T*_2_, *T*_3_	4.9–8.3

HCT [L/L]	0.40 ± 0.04	0.33–0.48	0.41 ± 0.04	0.36–0.5	0.33 ± 0.07 ^*∗∗∗*^ *T*_0_, *T*_1_	0.26–0.73	0.37 ± 0.04 ^*∗*^ *T*_0_, *T*_1_	0.27–0.45	0.40 ± 0.04 ^*∗∗*^ *T*_2_	0.30–0.46

MCV [fL]	83.7 ± 4.32	70–92	84.40 ± 3.74	77–92	84.32 ± 4.33	70–92	84.26 ± 4.7	68–90	83.27 ± 4.76	65–90

MCH [fmol]	1.55 ± 0.11	1.19–1.73	1.56 ± 0.08	1.38–1.72	1.87 ± 0.23 ^*∗∗∗*^ *T*_0_, *T*_1_	1.30–2.15	1.81 ± 0.13 ^*∗∗*^ *T*_0_, *T*_1_	1.21–1.79	1.46 ± 0.11 ^*∗∗*^ *T*_2_, *T*_3_	1.08–1.65

MCHC [mmol/L]	18.52 ± 0.39	17.1–19.1	18.51 ± 0.46	17.70–19.6	22.17 ± 2.09 ^*∗∗∗*^ *T*_0_, *T*_1_	15.60–24.2	19.15 ± 0.48 ^*∗∗∗*^ *T*_2_	17.7–20	17.52 ± 0.43 ^*∗∗*^ *T*_0_, *T*_1_, ^*∗∗*^ *T*_2_, *T*_3_	16.5–18.7

RDW [%]	12.86 ± 1.14	10.9–16.3	12.84 ± 0.89	11.20–15.6	15.11 ± 1.44 ^*∗∗∗*^ *T*_0_, *T*_1_	12.7–21	14.11 ± 0.99 ^*∗∗∗*^ *T*_0_, *T*_1_	12.90–17.2	13.65 ± 1.13 ^*∗∗∗*^ *T*_2_	12.4–17.6

WBC [10^9^/L]	7.45 ± 1.25	5.00–9.7	6.95 ± 1.26	4.60–9.8	6.91 ± 1.08	5.20–9.4	6.49 ± 1.26 ^*∗*^ *T*_0_	4.7–9.2	7.04 ± 1.48	4.8–10.4

LYM [10^9^/L]	2.59 ± 0.56	1.80–3.8	2.41 ± 0.57	1.60–3.7	2.51 ± 0.6	1.60–3.9	2.40 ± 0.66	1.4–4.5	2.45 ± 0.62	1.5–4.6

MON [10^9^/L]	0.51 ± 0.13	0.30–0.8	0.53 ± 0.21	0.20–1.2	0.47 ± 0.12	0.2–0.7	0.41 ± 0.13	0.2–0.7	0.46 ± 0.14	0.2–0.8

GRA [10^9^/L]	4.35 ± 1.04	2.60–6.2	4.00 ± 0.95	2.10–6.4	3.94 ± 0.86	2–5.8	3.68 ± 0.75	2.30–4.9	4.12 ± 1.19	2.9–7.5

PLT [10^9^/L]	248.64 ± 79.9	129–520	231.88 ± 58.41	120–345	208.52 ± 88.3	115–549	228.3 ± 59	126–357	252.89 ± 79.78	159–521

RBC: red blood cells; HB: hemoglobin; HCT: hematocrit; MCV: mean corpuscular volume; MCH: mean corpuscular hemoglobin; MCHC: mean corpuscular hemoglobin concentration; RDW: red blood cell distribution width; WBC: white blood cells; LYM: lymphocytes; MON: monocytes; GRA: granulocytes; PLT: thrombocytes.

^*∗*^Significance of differences at *p* ≤ 0.05 versus before.

^*∗∗*^Significance of differences at *p* ≤ 0.01 versus before.

^*∗∗∗*^Significance of differences at *p* ≤ 0.001 versus before.

**Table 4:** Biochemical indices changes following the exercise programme.

Parameters	*T*_0_		*T*_1_		*T*_2_		*T*_3_		*T*_4_	
	$\overset{-}{x}$	Min–max	$\overset{-}{x}$	Min–max	$\overset{-}{x}$	Min–max	$\overset{-}{x}$	Min–max	$\overset{-}{x}$	Min–max
Glucose [mg/dL]	73.34 ± 17.22	40.43–102.84	73.28 ± 17.80	36.88–111.35	72.57 ± 15.37	41.13–114.89	79.98 ± 26.09	43.97–174.82	79.11 ± 12.34	60.28–103.19

Uric acid [mg/dL]	2.25 ± 0.65	0.98–3.41	1.99 ± 0.56	1.10–3.29	2.17 ± 0.73	1.28–3.48	2.47 ± 0.64	1.52–4.57	2.11 ± 0.74	0.95–3.9

Cholesterol [mg/dL]	205.91 ± 37.64	143–287	207.32 ± 45.39	140–266	208.28 ± 38.29	151–279	189.60 ± 48.99 ^*∗*^ *T*_0_	122–253	226.73 ± 48.41 ^*∗*^ *T*_0_	110–302

HDL [mg/dL]	29.88 ± 5.27	21.24–41.59	28.57 ± 7.06	19.47–42.03	30.18 ± 6.7	19.47–42.57	31.97 ± 4.49	21.68–43.81	26.97 ± 4.49	21.68–43.81

LDL [mg/dL]	146.31 ± 32.76	90.2–238.8	130.23 ± 25.29 ^*∗*^ *T*_0_	66.19–166.68	131.84 ± 31.74 ^*∗*^ *T*_0_	93.84–233.38	132.37 ± 34.22 ^*∗*^ *T*_0_	72.08–253.42	153.10 ± 31.41	53.52–197.53

TG [mg/dL]	125.91 ± 56.71	37.68–297.1	108.58 ± 45.44 ^*∗*^ *T*_0_	57.97–220.29	111.59 ± 77.45 ^*∗*^ *T*_0_	34.78–314.49	112.45 ± 72.72 ^*∗*^ *T*_0_	57.97–437.68	130.21 ± 57.95	66.67–297.1

Adiponectin [ng/mL]	63.64 ± 30.42	16.37–124.1	62.86 ± 31.79	15.9–104.9	56.74 ± 27.99	14.97–113.7	63.88 ± 30.88	16.820–126.4	63.93 ± 31.37	20.3–139

Resistin [ng/mL]	4.25 ± 1.77	2.25–8.38	3.83 ± 1.46	2.01–8.38	4.24 ± 1.63	2.39–7.78	3.93 ± 1.54	1.96–7.98	3.80 ± 1.38	2.07–7.08

Leptin [pg/mL]	239.03 ± 116.67	45.68–508.2	168.05 ± 89.64	42.55–385.5	172.86 ± 115.09	24.95–559.9	189.13 ± 103.2	40.39–520.9	174.54 ± 100.56	50.39–411.2

Visfatin [ng/mL]	0.77 ± 0.5	0.03–2.18	1.05 ± 0.58	0.21–2.35	1.38 ± 0.23	0.19–2.09	1.47 ± 0.6 ^*∗∗∗*^ *T*_0_, ^*∗∗*^ *T*_1_, ^*∗*^ *T*_2_	0.56–2.73	1.37 ± 0.55 ^*∗∗∗*^ *T*_0_, *T*_1_, ^*∗∗*^ *T*_2_	0.50–5.74

^*∗*^Significance of differences at *p* ≤ 0.05 versus before.

^*∗∗*^Significance of differences at *p* ≤ 0.01 versus before.

^*∗∗∗*^Significance of differences at *p* ≤ 0.001 versus before.
